# Delay in seeking treatment and associated factors among pulmonary tuberculosis patients attending public health facilities in the Metekel zone, Benishangul Gumuz region, Western Ethiopia

**DOI:** 10.3389/fpubh.2024.1356770

**Published:** 2024-02-26

**Authors:** Yaregal Animut, Abera Birhanu Godno, Solomon Gedlu Nigatu, Saron Abeje Abiy

**Affiliations:** ^1^Department of Epidemiology and Biostatistics, Institute of Public Health, College of Medicine and Health Sciences, University of Gondar, Gondar, Ethiopia; ^2^Public Health Emergency Management Directorate, Benishangul Gumuz Regional Health Bureau, Assosa, Ethiopia; ^3^Department of Clinical Midwifery, School of Midwifery, College of Medicine and Health Sciences, University of Gondar, Gondar, Ethiopia

**Keywords:** patient delay, pulmonary tuberculosis, treatment, Western Ethiopia, associated factors

## Abstract

**Background:**

Tuberculosis is a major global public health problem and a leading cause of morbidity and mortality in Ethiopia. TB prevention and control in low-income countries, such as Ethiopia, face significant challenges, including late detection and treatment initiation. A delay in the initiation of tuberculosis treatment increases the morbidity and mortality of patients and community transmission. Therefore, this study aimed to assess patient delay and associated factors among pulmonary tuberculosis patients attending public health facilities in the Metekel Zone, Benishangul Gumuz Region, Western Ethiopia.

**Methods:**

An institution-based cross-sectional study was conducted from March to August 2020 among newly diagnosed pulmonary tuberculosis patients. All pulmonary tuberculosis patients (416) who came to all public health facilities of the Metekel zone for treatment during the period were included. Data were collected through face-to-face interviews using a structured and pretested questionnaire. A multivariable logistic regression was fitted to identify independent factors for delay in seeking treatment among PTB patients. Adjusted odds ratios with 95% CIs were determined, and variables with *p* values <0.05 were considered statistically significant.

**Results:**

Nearly three-fourths 302 (72.6, 95% CI: 68.5, 76.7) of the patients were delayed in seeking medical advice, with a median patient delay of 27 days (IQR: 21–31). Age of the patients [above 54 years (AOR = 2.65, 95% CI: 1.30, 5.40), 36–54 years (AOR = 1.86, 95% CI: 1.14,3.02)], family size of 5 members and above (AOR = 1.62, 95% CI: 1.10–3.14), travel time above 60 min (AOR = 3.65, 95% CI: 1.55, 8.60), history of visits to informal care providers (AOR = 1.74, 95% CI: 1.11, 3.14), and poor knowledge about PTB (AOR = 1.64, 95% CI: 1.04–2.44) were statistically significant factors associated with delays in seeking treatment among PTB patients.

**Conclusion:**

Most pulmonary tuberculosis patients delay seeking medical advice for their illnesses. Delays in seeking treatment were associated with older age, large household size, longer travel time to reach the nearby health facility, visiting informal care providers, and poor knowledge about pulmonary tuberculosis. Hence, it is crucial to consider community screening programs, enhance public awareness, and ensure the accessibility of TB diagnostic and treatment services.

## Background

Tuberculosis (TB) is an infectious bacterial disease caused by the bacillus *Mycobacterium tuberculosis*. It primarily affects the lungs (pulmonary TB), accounting for 85% of all TB cases, but it can also affect other parts of the body (1). It is a major cause of morbidity, ranking among the top 10 causes of death worldwide and being the leading cause of death from a single infectious agent (2).

TB is a significant public health problem, with an estimated one-quarter of the global population being infected with tubercle bacilli and therefore at risk of developing active disease (3). According to the 2019 WHO report, approximately 10.0 million people globally (132 cases per 100,000 population) fell ill with TB in 2018. In Africa, the estimated total incidence and HIV-negative TB mortality were 231 and 37 per 100,000 population, respectively. Ethiopia is one of the top 20 high-burden countries for TB, TB/HIV, and MDR-TB, with an estimated annual incidence of TB and HIV-negative TB mortality of 151 and 22 per 100,000 populations, respectively (4).

Achieving the goal of eradicating the TB epidemic by 2030 is one of the health targets outlined in the United Nations Sustainable Development Goals (SDGs) (5). The End TB Strategy focuses on actions needed to accelerate progress in providing access to TB diagnosis and treatment in each country. It emphasizes integrated patient-centered care and prevention, along with early diagnosis and prompt treatment within 2 weeks of the onset of signs and symptoms (6). Ethiopia has officially supported the global “END TB strategy” and aligned the National TB Strategic Plan with the National Health Sector Transformation Plan. The National End TB Strategy aims to reduce TB-related mortality by 95% and new TB cases by 90% between 2015 and 2035. The program is dedicated to enhancing accessibility and fairness in TB services for vulnerable and marginalized populations, as these groups often face socioeconomic and legal obstacles that result in delays in receiving appropriate care (7).

Ensuring early detection and timely initiation of treatment is crucial for an effective tuberculosis control program. Delays in diagnosis and treatment remain a challenge in TB prevention and control programs. Delays in the initiation of tuberculosis treatment pose various risks, including unfavorable treatment outcomes, adverse social and economic consequences, increased transmission of TB, and reduced productivity and social stability (7, 8).

Studies in different regions of Ethiopia indicate that late patient health-seeking behavior is the major contributor to treatment delay in tuberculosis treatment (8–10). The magnitude of patient delay among pulmonary tuberculosis patients ranges from 30 to 90%, with a median delay of 17 days to 45 days (9–15). Patient delay can be associated with various factors that either promote or impede health-seeking behavior. Previous studies in different areas of Ethiopia have identified factors such as older age (9, 16), lower educational status (10, 17), rural residence (10, 18), low monthly income (15, 18), large family size (9), long distance from the health institution (10, 11), lack of knowledge about tuberculosis (12, 15), self-medication practice (9, 14), prior visit to informal providers (14, 15, 18), and low perceived severity of the illness (10, 15).

The TB case detection rate in the Metekel zone of Benishangul Gumuz Regional State, West Ethiopia, was 36% in 2015, which is significantly lower than the minimum national target of 70% (19). The low case detection rate could be attributed to patients’ delay in seeking health care for their TB symptoms. Therefore, it is crucial to conduct a study on patients’ delay in seeking treatment and the associated factors among pulmonary TB patients.

## Methods and materials

### Study design and period

A multicenter institution-based cross-sectional study was conducted from March 10 to August 10, 2020.

### Study setting

The study was conducted in the Metekel Zone, Benishangul Gumuz Regional State public health facilities. The Benishangul Gumuz region is located in the western part of the country and is bordered by the Amhara region in the northeast, the Oromia region in the southeast, and Sudan in the west. The Mekel zone is one of the three administrative zones in the region. It comprised seven districts and a one-city administration. The zonal capital, Gilgel Belese town, is situated 446 km west of Addis Ababa. The total population of the zone, as based on the 2007 census projection in 2020, is approximately 468,353, of which 49% were male and 95% were living in rural areas. Agriculture and grazing are the main sources of livelihood in the zone. In the zone, there are 3 public hospitals and 16 public health centers that provide tuberculosis laboratory diagnosis and DOT services to the community. A total of 1,100 health care professionals were working in these institutions, and a total of 825 PTB patients were reported in the zone in 2019.

### Population and sample

All adult clients diagnosed with PTB at the DOTs unit of public health facilities of the Metekel zone during the study period were the study population. The sample size of the study was calculated using a single population proportion formula [*n* = (*Z*α/2)2 × *p*(1 − *p*)/*d*2] with the following assumptions: the proportion of PTB patients who were delayed in seeking health care from the previous study (53.4%) (16), the probability of type 1 error or alpha 0.05, and the 95% confidence level. The calculated sample size was 382, and the final sample size used in this study was 420 considering a 10% nonresponse rate.

From 16 health centers and 3 hospitals that provide tuberculosis diagnosis and DOT services in the Metekel Zone, 3 health centers were not included in this study due to insecurity in the areas at the time of data collection. All newly diagnosed PTB patients from each DOT center of the 16 public health facilities (3 hospitals and 13 health centers) of the Metekel Zone during the study period were included in the study. Accordingly, the following samples were included from each DOTs center: Mankush HC (34), Dibati HC (30), Gublak HC (18), Manbuk HC (30), Gilgel Beles HC (34), Mender 14 HC (15), F/Selam HC (30), Berber HC (22), Galesa HC (24), Bullen HC (30), Dobi HC (16), Gesgesa HC (15), Debre Zeit HC (32), Pawe Hospital (40), Bullen Hospital (22), and Wombera Hospital (24).

### Variables of the study

*Dependent variable*: Delay in seeking treatment (Delayed or Not Delayed).

*Independent variables*:

*Sociodemographic variables*: Age, sex, marital status, residence, educational status, household size, occupation, family income, and time to reach the nearest health facility.

*Clinical and behavioral variables*: Status of a patient at the first visit, comorbidity, contact history to TB patient, history of a visit to informal care providers, types of treatment center, history of smoking, alcohol drinking status, and knowledge about TB.

### Operational definition

#### Delay in seeking treatment

This is the time interval between the onset of patient-recognized PTB symptom(s) and consultation with a healthcare provider. TB patients who consulted a formal health care provider longer than 21 days (2 weeks entry point for presumptive TB case and 1 week period to seek health care) after the onset of initial constitutional signs and symptoms of TB were considered delayed (11, 20, 21).

#### Knowledge of TB

Six item questions about knowledge of TB (causes, symptoms, mechanism of transmission, curability, the existence of vaccine) were used, and variables measuring knowledge were recorded on a 3-point Likert scale (0–worst and 2-best). We assessed the reliability of items used to measure knowledge about TB, and we found a Cronbach alpha of 0.73. Patients who scored more than average were considered to have good knowledge, and those who scored less than average (50%) were considered to have poor knowledge (11, 22).

Informal healthcare providers are drug shops/vendors, traditional injectors/healers, religious institutions, etc. (23).

#### Smear-positive PTB

A person who has at least one positive result on AFB microscopy or a person whose Xpert MTB/RIF test result detected mycobacteria with susceptibility to rifampicin (24).

#### Smear-negative PTB

Patient with symptoms suggestive of TB, with at least two sputum specimens that were negative for AFB by microscopy and in whom Xpert MTB/RIF test results detect no *mycobacterium*; the decision to empirically treat with a full course of Anti-TB regimen is made with the help of evidence from supporting tests and with aid of sound clinical decision (24).

### Data collection tools and procedure

An interviewer-administered structured questionnaire adapted from previous studies (8, 25) was used to collect sociodemographic, clinical, behavioral, and other variables related to TB treatment delay. The self-reported date of onset for the illness was taken as the date of onset of signs and symptoms for tuberculosis. Cough was taken as the main symptom, followed by nonspecific systemic symptoms such as chest pain, fever, night sweats, shortness of breath, and loss of weight taken as the onset of illness, whichever came first used to count the date of patient care seeking.

At each study site, we recruited a nurse with a degree in nursing and fluent in Amharic, and local languages such as Gumuz and Shinashigna, as data collector Data collectors and supervisors were trained for 1 day on the objective of the study and the basics of interviewing before the actual data collection. The questionnaire was first prepared in English and then translated into Amharic and back to English by language experts to check consistency and conceptual similarity, and it was also pretested at Assossa Health Center. The data collection process was closely monitored by the principal investigator and the supervisors throughout the data collection period. Filled questionnaires were checked regularly for completeness of information, and any problems were immediately discussed with the data collectors.

### Data processing and analysis

Data were entered into Epi info version 7 and exported to SPSS version 20 software for analysis. Frequency distribution tables and summary measures such as percentages and medians were used to describe the data. A binary logistic regression model was fitted to identify factors associated with delays in seeking treatment among pulmonary tuberculosis patients. Bivariable analysis was carried out for all independent variables with an outcome variable, and variables with a *p* value ≤0.2 were entered into a multivariable logistic regression model to identify the independently associated factors of delay in seeking treatment for PTB. The adjusted odds ratio with 95% CI was determined, and variables with a *p* value ≤0.05 were considered statistically significant.

## Results

### Sociodemographic characteristics of the study participants

A total of 416 newly diagnosed pulmonary tuberculosis patients were included in the study, with a response rate of 99.5%. The mean age of the study participants was 40 years (SD ± 14 years), and more than half 232 (55.8%) of the patients were males. Nearly two-thirds (268, 64.4%) of the study subjects resided in rural areas. Approximately 161 (38.7%) were farmers by occupation, and 94 (22.6%) patients were not able to read and write. More than half (241, 57.9%) of the participants traveled on foot to the nearest health facility, and 165 (39.7%) reported that it took more than half an hour to reach the nearest health facility (Table 1).

**Table 1:** Sociodemographic characteristics of PTB patients attending public health facilities in the Metekel Zone, West Ethiopia, 2020 (*n* = 416).

Variables	Frequency (*n*)	Percentage (%)
Sex		
Male	232	55.8
Female	184	44.2
Age category		
≤35	177	42.5
36–54	167	40.2
≥55	72	17.3
Residence		
Rural	268	64.4
Urban	148	35.6
Household size		
≤5	311	74.8
>5	105	25.2
Marital status		
Single	103	24.8
Married	238	57.2
Divorced	30	7.2
Windowed	45	10.8
Educational level		
Unable to read and write	94	22.6
Primary (1-8)	211	50.7
Secondary (9-12)	69	16.6
College and above	42	10.1
Occupation		
Farmer	161	38.7
Housewife	38	9.1
Student	40	9.6
Merchant	103	24.8
Employed	74	17.8
Monthly income		
≤500 Birr	137	32.9
501–1,000 Birr	95	22.8
>1,000 Birr	184	44.2
Transport method		
Vehicle	175	42.1
On foot	241	57.9
Total traveled time		
≤30 min	251	60.3
31–60 min	109	26.2
>60 min	56	13.5

### Clinical and behavioral characteristics of respondents

Of the total participants, 309 (74.3%) were smear-positive, and the majority (407, 97.8%) of patients presented with persistent cough followed by shortness of breath (84, 20.2%). One-third (133, 32%) of the respondents had a history of contact with previous TB patients. Approximately 121 (27%) had comorbidities, of which HIV/AIDS accounted for 49 (40.4%), diabetes accounted for 36 (29.8%), and hypertension accounted for 36 (29.8%). More than two-thirds 283 (68%) of the respondents had poor knowledge of TB. Although most of the study participants (347, 83.4%) had heard about TB before, approximately 330 (79.3%) of the respondents did not know microbes as the cause of TB disease. Nearly half (202, 48.6%) of the participants heard information about TB from their friends or relatives. The majority of respondents did not know about the symptoms (238, 57.2%) and contagiousness (267, 64.2%) of TB. Of the total participants, 18 (4.3%) were smokers, and 83 (20%) were current alcohol drinkers (Table 2).

**Table 2:** Clinical and behavioral characteristics of PTB patients attending public health facilities in the Metekel Zone, West Ethiopia, 2020 (*n* = 416).

Characteristics	Frequency (*n*)	Percentage (%)
Smear positivity		
Smear positive	309	74.3
Smear negative	107	25.7
Symptoms at presentation		
Cough	407	97.8
Shortness of breath	84	20.2
Fever	59	14.2
Weight loss	53	12.7
Chest pain	30	7.2
Night sweating	14	3.4
Types of treatment center		
Hospital	86	20.7
Health center	330	79.3
History of visit to informal care providers		
Yes	276	66.3
No	140	33.7
Status of a patient at first contact		
Working	185	44.5
Ambulatory	184	44.2
Bedridden	47	11.3
Previous contact with TB patient		
Yes	133	32.0
No	283	68.0
Comorbidity		
Yes	121	29.1
No	295	70.9
History of alcohol drinking		
Nonconsumer	198	47.5
Previous consumer	135	32.5
Current consumer	83	20.0
History of smoking		
Nonsmoker	356	85.6
Previous smoker	42	10.1
Current smoker	18	4.3
Knowledge about TB		
Good Knowledge	133	32.0
Poor knowledge	283	68.0

#### Proportion of pulmonary tuberculosis patients who delayed seeking treatment

Nearly three-fourths 302 (72.6, 95% CI: 68.5, 76.7) of the patients were delayed in seeking medical advice, which means they visited health institutions 21 days after the onset of their illness. Two-thirds 276 (66.3%) of the participants had a history of visits to informal health care providers for the current symptoms before they came to the current health facilities from which 90 (21.6%) visited drug vendors. The median patient delay was 27 days (IQR: 21–31), with minimum and maximum delays of 10 and 120 days, respectively.

#### Factors associated with delay in seeking treatment among pulmonary tuberculosis patients

In this study, age, family size, time to reach the nearest health facility, and knowledge about PTB were significantly associated with delays in seeking treatment among PTB patients.

The odds of delay in seeking treatment for PTB were 2.65 times higher (AOR = 2.65, 95% CI: 1.30, 5.40) among patients aged above 54 years and 1.86 times higher (AOR = 1.86, 95% CI: 1.14,3.02) among patients aged 36–54 years than among patients aged less than 35 years. Tuberculosis patients with family sizes of 5 and above were 1.62 times more likely (AOR = 1.62, 95% CI: 1.10–3.14) to delay seeking treatment than those with a family size of fewer than 5 members. The odds of delay in seeking treatment among PTB patients who traveled above 60 min to reach a nearby health facility were 3.65 times higher (AOR = 3.65, 95% CI: 1.55, 8.60) than those who traveled less than 30 min. Patients who had a history of visits to informal care providers were 1.74 times more likely (AOR = 1.74, 95% CI: 1.11, 3.14) to delay seeking treatment from formal care providers than their counterparts. Tuberculosis patients who had poor knowledge about PTB were 1.64 times more likely (AOR = 1.64, 95% CI: 1.04–2.44) to delay seeking treatment than patients with good knowledge (Table 3).

**Table 3:** Factors associated with patients’ delay in PTB treatment in public health facilities in the Metekel Zone, West Ethiopia, 2020 (*n* = 416).

Variables	Patient delay			
	Delayed	Not delayed	COR (95% CI)	AOR (95% CI)
Residence				
Rural	203	65	1	1
Urban	99	49	1.55 (0.99–2.41)	0.73 (0.41–1.29)
Age in years				
≤35	112	65	1	1
36–54	130	37	2.04 (1.27–3.28)*	1.86 (1.14–3.02)*
>54	60	12	2.90 (1.45–5.79)**	2.65 (1.30–5.40)**
Household size				
<5	217	94	1	1
≥5	85	20	1.84 (1.07–3.17)*	1.62(1.10–3.14) *
Occupation				
Farmer	126	35	1	1
Housewife	26	12	0.60 (0.28–1.31)	0.82 (0.35–1.94)
Student	25	15	0.46 (0.22–0.97)*	0.83 (0.34–2.02)
Merchant	74	29	0.71 (0.40–1.25)	1.02 (0.53–1.98)
Employed	51	23	0.59 (0.24–1.41)	0.82 (0.35–1.95)
Types of transportation				
On foot	182	59	1.41(0.92–2.17)	1.96(0.98–3.45)
By Vehicle	120	55	1	1
Time to reach the nearby health facility				
≤30 min	167	84	1	1
31–60 min	86	23	1.88 (1.11–3.19)*	1.71 (0.99–2.95)
>60 min	49	7	3.52 (1.53–8.11)**	3.65 (1.55–8.60)**
History of visit to informal care providers				
Yes	211	65	1.75(1.12–3.22)*	1.74(1.11–3.14)*
No	91	49	1	1
Knowledge				
Poor knowledge	214	69	1.59(1.05–2.5)*	1.64(1.04–2.44)*
Good knowledge	88	45	1	1
Alcohol drinking				
Nonconsumer	130	68	1	1
Ex-consumer	110	25	2.30 (1.36–3.89)*	0.68 (0.37–1.26)
Current consumer	62	21	1.54 (0.87–2.75)	1.23 (0.61–2.49)

* = statistically significant at *p* value 0.05, ** = statistically significant at *p* value 0.001.

## Discussion

In the current study, the majority (72.6%) of patients were delayed in seeking medical advice beyond the WHO-recommended time, which is 21 days since they first experienced symptoms of tuberculosis (25). This finding is consistent with a nationwide study conducted in Ethiopia 72.3% (26), Northwest Ethiopia (68.7%) (27), India (73.7) (28), and Tanzania (71%) (29). However, it is higher than studies conducted in other parts of the country, such as Bahirdar (62%) (30), Hadiya (58.2%) (30), North Shewa (59.9%) (12), and Addis Ababa (42.1%) (11), as well as studies in Zimbabwe (48%) (31), Kenya (65.4%) (32), Nigeria (42%) (33), and Ghana (60.3%) (34). This implies that a large proportion of TB patients continued to serve as reservoirs of infection and would have continued to transmit the disease in the community.

In our study, the median patient delay was 27 days. This finding is consistent with studies conducted in other parts of Ethiopia (13, 20, 30, 35), Zimbabwe (31), Kenya (36), and Uganda (37), which revealed a median patient delay of 25–30 days. However, the median patient delay in our study was higher than that in studies in different areas of Ethiopia (17–21 days) (11, 14, 26, 28) and Nigeria (8 days) (39). This difference may be due to sociocultural differences between study participants since our study was conducted in one of the developing regional states of the country, Benishangul Gumuz. In our study, two-thirds of the study participants visited informal health care providers such as religious institutions, self-treatment from drug vendors and traditional healers before seeking care at public health institutions, which contributed to a higher median patient delay. This finding highlights the need to improve patients’ health-seeking behavior by raising community awareness about the importance of the early recognition of PTB symptoms.

Furthermore, patients older than 54 years and patients aged 36–54 years were more likely to experience prolonged delays in seeking treatment compared to patients under 35 years. This finding is consistent with studies conducted in Ethiopia (9, 16, 30, 38), Nigeria (39), Portugal (40), and Tanzania (29). This may be related to the fact that older people may depend on others, making it difficult for them to visit health facilities at their preferred time. Patients between the ages of 36 and 54 are productive and independent but are also more likely to have larger family sizes than patients under the age of 35, which can burden them with other family matters and delay their visits to healthcare facilities for their symptoms.

The time taken to reach a health facility was a significant factor associated with delays in seeking treatment among PTB patients. Patients who traveled more than an hour to reach a nearby health facility had a threefold increased delay in seeking treatment compared to those who traveled less than 30 min. This finding is supported by studies from different parts of Ethiopia (11, 41), China (42), Italy (43), Nigeria (39), and Nepal (44). This might be due to the difficulties in accessing health facilities, and it is crucial to utilize health extension workers to improve the detection of presumed tuberculosis cases at the community level.

Tuberculosis patients with a family size of five members or more were more likely to delay seeking treatment compared to those with smaller families. This finding is consistent with studies conducted in the Eastern and Southern parts of Ethiopia (9, 13). A larger family size may lead to increased responsibilities and financial constraints, which can hinder timely visits to healthcare facilities for their illnesses.

Patients who had a history of visits to informal care providers were nearly twice as likely to delay seeking treatment from formal care providers as their counterparts. This finding is consistent with previous studies in Ethiopia (14–16, 38, 41), Mozambique (45), and Zimbabwe (31). In our study, two-thirds of the patients initially sought care from informal providers before coming to health institutions. This indicates a higher preference for informal treatments among TB patients, which may increase the risk of community transmission and the severity of the illness. Therefore, increasing awareness about the disease and utilizing the aid of informal health care providers, such as traditional or religious healers and drug vendors, in the referral process can help in the early identification of suspected TB cases and prompt linkage to DOT centers.

Knowledge about tuberculosis was also found to be an important factor associated with delays in seeking treatment. Patients with poor knowledge about PTB were 1.64 times more likely to delay seeking treatment than patients with good knowledge. This finding is similar to studies conducted in other parts of Ethiopia (15, 16, 20, 38), Nigeria (33), and Mozambique (45). This demonstrates how patients with limited understanding of the signs and natural course of the disease thought that their symptoms would eventually go away or that they would seek out alternative therapies from informal providers such as traditional or religious healers or engage in self-medication with over-the-counter medications, leading to delays in seeking medical advice.

In light of our findings, it is imperative to consider strategic interventions aimed at reducing delays in seeking treatment among pulmonary TB patients. Implementing targeted public health campaigns could play a pivotal role in enhancing public awareness regarding the importance of early TB detection and timely treatment (46). Community engagement, especially involving community leaders, is instrumental in reshaping healthcare-seeking behaviors (47). Establishing collaborative efforts with community leaders can facilitate the dissemination of accurate information about TB, dispelling myths, and encouraging individuals to seek formal medical care promptly (48). The introduction of community screening programs is another key strategy to address delays in seeking treatment (49). By bringing TB testing and treatment closer to communities, these programs can overcome barriers related to geographical distance and contribute to early case detection.

### Limitation of study

One potential limitation of this study is the possibility of recall bias, as patients may not accurately estimate or remember the exact date of onset of their illness. Additionally, this study may not be generalizable to all pulmonary tuberculosis patients since it did not include participants from private health facilities.

## Conclusion

The majority of pulmonary tuberculosis patients delay seeking medical advice for their illnesses. Factors associated with these delays included older age, large household size, longer travel time to reach the nearby health facility, visits to informal care providers, and poor knowledge about pulmonary tuberculosis. Therefore, it is important to strengthen community awareness activities, introduce community screening programs, and ensure the accessibility of TB diagnostic and treatment services.

## Data availability statement

The original contributions presented in the study are included in the article/supplementary material, further inquiries can be directed to the corresponding author.

## Ethics statement

The studies involving humans were approved by Institutional Review Board (IRB) of the Institute of Public Health, University of Gondar, Ethiopia. The studies were conducted in accordance with the local legislation and institutional requirements. The ethics committee/institutional review board waived the requirement of written informed consent for participation from the participants or the participants' legal guardians/next of kin because since the study was an observational study based on interviews it doesn't have any risk, But we obtained informed verbal consent.

## Author contributions

YA: Conceptualization, Data curation, Formal analysis, Investigation, Methodology, Software, Supervision, Validation, Writing – original draft, Writing – review & editing. AG: Conceptualization, Data curation, Formal analysis, Investigation, Methodology, Software, Supervision, Validation, Writing – review & editing. SN: Formal analysis, Investigation, Methodology, Software, Supervision, Writing – review & editing. SA: Formal analysis, Investigation, Methodology, Supervision, Writing – original draft, Writing – review & editing.
